# In Search of the Optimal Conditions to Process Shape Memory Alloys (NiTi) Using Fused Filament Fabrication (FFF)

**DOI:** 10.3390/ma13214718

**Published:** 2020-10-22

**Authors:** Pedro Carreira, Fábio Cerejo, Nuno Alves, Maria Teresa Vieira

**Affiliations:** 1 CDRSP—Centre for Rapid and Sustainable Product Development, Polytechnic Institute of Leiria, Rua General Norton de Matos, Apartado 4133, 2411-901 Leiria, Portugal; 2 IPN—Instituto Pedro Nunes, Rua Pedro Nunes, 3030-199 Coimbra, Portugal; fcerejo@ipn.pt; 3 CEMMPRE—Centre for Mechanical Engineering, Materials and Processes, University of Coimbra, Pinhal de Marrocos, 3030-788 Coimbra, Portugal

**Keywords:** nickel–titanium (NiTi), shape memory alloys (SMA), fused filament fabrication (FFF), additive manufacturing (AM), powder filaments, shaping, debinding and sintering (SDS)

## Abstract

This research was performed so as to investigate the additive manufacturing of NiTi shape memory alloys, which is associated with direct processes, such as selective laser melting. In addition to its expensive production costs, NiTi readily undergoes chemical and phase modifications, mainly as a result of Ni loss during processing as a result of high temperatures. This research explores the potential usefulness of NiTi as well as its limitations using indirect additive processes, such as fused filament fabrication (FFF). The first step was to evaluate the NiTi critical powder volume content (CPVC) needed to process high-quality filaments (via extrusion). A typical 3D printer can build a selected part/system/device layer-by-layer from the filaments, followed by debinding and sintering (SDS), in order to generate a near-net-shape object. The mixing, extruding (filament), printing (shaping), debinding, and sintering steps were extensively studied in order to optimize their parameters. Moreover, for the sintering step, two main targets should be met, namely: the reduction of contamination during the process in order to avoid the formation of secondary phases, and the decrease in sintering temperature, which also contributes to reducing the production costs. This study aims to demonstrate the possibility of using FFF as an additive manufacturing technology for processing NiTi.

## 1. Introduction

NiTi belongs to a class of materials named shape memory alloys (SMA), which are defined as any material, metallic or non-metallic, with the ability to restore its previously defined shape when exposed to a specific thermal cycle, through either the shape memory effect or superelasticity [1]. These alloys are widely used for engineering and medical applications, and have been expanded to other fields. However, they are known to be extremely difficult to process [2]. In fact, production based on NiTi as the master material is quite difficult, as the presence of microstructural defects or variation in the chemical composition can alter its transformation temperatures significantly [2,3]. For instance, varying the nickel content in NiTi by 1 at % gives rise to an Ms deviation of 100 K [4]. These modifications typically occur as a result of the presence of impurities and/or the formation of new intermetallic phases.

A common technology in additive manufacturing (AM) for processing NiTi powder particles is selective laser melting (SLM) [5,6,7], which involves higher temperatures than those needed for NiTi melting (1310 °C). The high temperatures produce a melt pool that is very susceptible to contamination by impurities, and tends to form undesired phases during rapid and uncontrolled cooling. Moreover, these high temperatures are difficult to control and, in general, allow significant chemical and phasic modifications that differ from NiTi. High temperatures in the melt pool can also induce nickel evaporation (Ni = 2913 °C), causing an imbalance in the NiTi chemical composition and/or phases present, consequently changing the transformation temperatures [8,9,10,11]. These problems have been the main reasons for the unsuccessful processing of NiTi via direct processes, such as SLM.

The indirect additive process of fused filament fabrication (FFF) is based on the additive manufacturing of polymers (fused deposition modelling (FDM)) and powder extrusion process/injection moulding (PEP/PIM). In these technologies, an optimized feedstock, in which metal powder particles are the raw material, is mixed with polymers (binder and additives). At present, in FFF technology, it is necessary to first produce the filament in order to obtain the 3D body shaping. However, other techniques, such as debinding and sintering (SDS), are needed for the final product consolidation [12,13]. The use of FFF solves some problems associated with direct processing, including nickel evaporation, although other difficulties remain. Prealloyed NiTi powders are the most popular for NiTi powder fabrication because of the high reactivity of the Ni and Ti elemental powders with the components of the binder during debinding and sintering. These steps are essential for the quality and final price of the products manufactured by FFF. Enhancing the reduction behaviour of the environmental atmosphere and decreasing the temperature and holding time of the sintering step may improve the implementation of the FFF process when the raw material is a prealloyed NiTi. The temperatures used during debinding and sintering may cause an imbalance in the Ni:Ti ratios and contribute to secondary phase formation. An Ni content above 50.5 at % promotes the formation of Ni-rich phases, which shift the Ni:Ti ratios. Moreover, the presence of contaminants, particularly O_2_ trapped inside the specimens and C resulting from the ustulation of the binder, also promotes the formation of undesired phases, such as TiC and Ni_2_Ti_4_O_x_, changing the Ni:Ti ratio and, consequently, the transformation temperatures. Table 1 summarizes the phases present, where PIM was used as the shaping process, after debinding and under different sintering conditions of prealloyed NiTi powder particles. These studies were performed between 2003 and 2016, and no heat treatment was able to retain NiTi as a single phase. Different temperatures and holding times, different atmospheres for sintering, and different binders were used with or without post-treatments.

The main objective of the present study is to produce near-net-shape 3D parts/systems/devices from prealloyed NiTi powder particles via SDS, where shaping is performed via layer-by-layer deposition by FFF from homemade filaments, and thermal debinding and sintering occur at the lowest temperatures and in a H_2_ atmosphere. Furthermore, filaments from the NiTi prealloyed powder are developed and optimized for FFF. Finally, this study highlights the effect of a reducing gas atmosphere (H_2_) and low-temperature sintering in the final phase composition of the NiTi 3D components.

## 2. Materials and Methods

**Figure 1:**
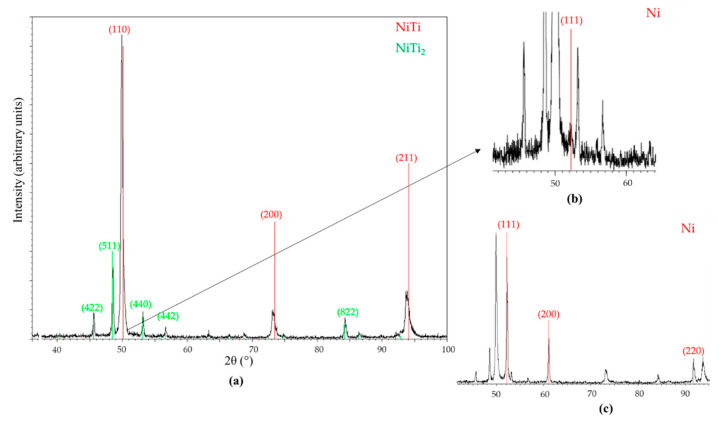
X-ray diffractogram of prealloyed NiTi powder containing (**a**) NiTi and NiTi_2_, (**b**) Ni, and (**c**) Ni after magnetic separation.

Prealloyed NiTi powder particles were supplied by LPW Technology Ltd [14]. The density of the powder was 6375 kg/m^3^ (after magnetic separation, that is, without loose Ni particles (8908 kg/m^3^)), and its specific surface area was 293.4 m^2^/kg. The bulk density of NiTi is 6450 kg/m^3^; a density that differs from that of the powder used may result from the variable content of one or more phases in the prealloyed powder, which can include a second phase that occurs during the synthesis of NiTi. In fact, structural analysis by X-ray diffraction (Malvern Panalytical, Egham, UK) of the prealloyed powder revealed phases other than NiTi and Ni; it also included NiTi_2_ (5640 kg/m^3^; Figure 1).

The density of the metallic powder was measured using Micromeritics Accupyc 1330 (Micromeritics Instruments Corporation, Norcross, GA, USA), and the particle size distribution (PSD) was evaluated using laser diffraction spectrometry (LDS, Malvern Panalytical, Egham, UK) with a Malvern Mastersizer 3000 instrument. The phase analysis was performed using a Philips X’Pert diffractometer (Egham, UK) at 40 kV with Bragg–Brentano geometry (θ–2θ), a cobalt anticathode (λ(k_α1_) = 0.178897 nm and λ(k_α2_) = 0.179285 nm), a current intensity of 35 mA, and a step rate of 0.25° s^−1^.

**Figure 2:**
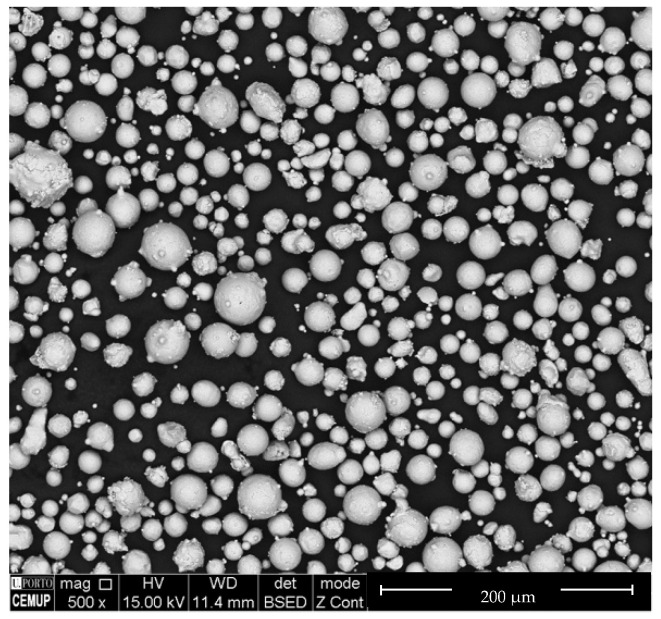
Shape of prealloyed NiTi powder particles.

**Figure 3:**
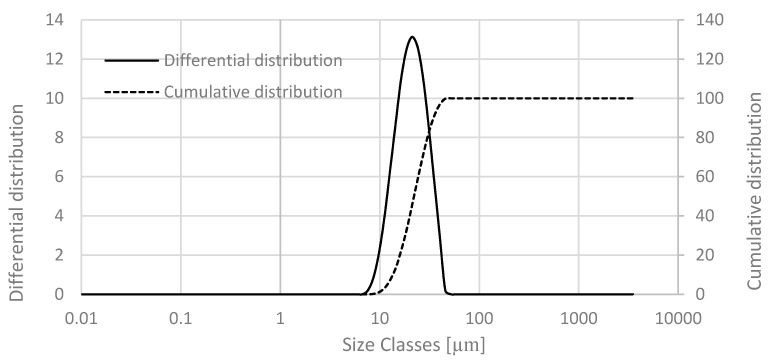
Particle size distribution of prealloyed NiTi powder.

The NiTi powder particles have a shape factor close to 1 (Figure 2) [19,20]. The particle size was d_50_ = 22.1 µm (Figure 3), which is typical of powder particles used in direct additive processes (i.e., SLM and electron beam melting (EBM)), but they could contribute to problems during sintering in indirect processes.

The homemade NiTi filament included prealloyed metallic particle powder and a master binder (M1) composed of polyolefin waxes and ethylenic polymers (Atect^®^ Company, Higashiomi, Japan). Moreover, in order to achieve suitable filament flexibility for storage and use in the 3D printer, other polymeric additives were added to the filament feedstock, including a thermoplastic elastomer (TPE) and a plasticizer (P). The optimization of the NiTi filament master binder and additive content was performed using the methodology for powder injection moulding feedstocks by evaluating the critical powder volume concentration (CPVC) [21,22,23]. The mixture of NiTi powder particles, master binder, and additives was optimized in a torque rheometer (Plastograph Brabender GmbH and Co., Duisburg, Germany) with a selected rotation blade speed of 30 rpm at a temperature of 180 °C. Afterward, the feedstock was granulated for use in extrusion filament equipment (Brabender GmbH and Co. E 19/25 D, Duisburg, Germany, without a calibration system) with a nozzle diameter of 1.75 mm (typical filament diameter of FFF printers) and a temperature of 180 °C. The mechanical characterization was performed by tensile and flexural tests on 20 filament specimens, 25 mm in length, that were randomly removed from the filament spool. The filaments were mechanically tested with a TA.XTplusC texture analyser (Stable Micro Systems, Godalming, UK) at a speed of 0.5 mm s^−1^ at room temperature (22 °C). 

NiTi objects, formed layer-by-layer from filaments, were three-dimensionally (3D) modelled and translated into a stereolithography (STL) file using Solidworks software (premium research 2019–2020) from Dassault Systèmes [24], and the G-Code was created using the CURA software (V4.4.1) from Ultimaker B.V. [25]. The printer (Hephestos2 from BQ, Madrid, Spain) had different nozzle diameters of 0.2, 0.4, and 0.8 mm.

The two-step consolidation of the green body (powder particles that are shaped but not sintered) parts/systems/devices was performed, which involved thermal debinding and sintering. The thermal debinding cycle must eliminate all organic binders and additives. However, the elimination of these components should proceed in a step-by-step manner so as to avoid changing the selected geometry. Thus, it was necessary to evaluate the temperatures corresponding to weight loss at different steps of feedstock component degradation/ustulation. Therefore, debinding was performed based on the thermal gravimetric analysis (TGA Q500, TA instruments, New Castle, DE, USA) of the master binder and additives in a non-oxidant dynamic atmosphere (N_2_) at a heating rate of 10 °C min^−1^. After debinding, the brown body (specimens after debinding and before sintering) was sintered at different temperatures and holding times (1100 °C (1 h), 1165 °C (1 and 5 h)). The sintering step was performed at a heating and cooling rate of 5 °C min^−1^.

After polishing, all of the specimens were observed using optical microscopy (OM) and scanning electron microscopy (SEM; FEI Quanta 400 FEG ESEM/EDAX Genesis, Thermo Fisher Scientific, Waltham, MA, USA).

Ultramicrohardness equipment from Fisher Instruments (Fischerscope H100, Waltham, MA, USA) was used. Nominal loads within the range of 4–1000 mN can be applied, with a resolution greater than 1 mN. The indenter was Vickers. The indentation depth was measured using a capacitance displacement gauge with an accuracy of 2 nm. During the test, the load was increased in steps until the nominal test load was reached. The number of steps and the time between them were selected before the test; the first load step was always equal to 0.4 mN. Each of the four specimens was tested 40 times.

The major novelty presented in this work is the use of FFF to process NiTi prealloyed powder particles. Moreover, two other goals that should be attained is to test the influence of 100% hydrogen atmosphere during debinding and sintering, in order to avoid the presence of oxides and carbides. The second goal is to evaluate the differences in decreasing the temperature of sintering than that typically refereed in the bibliography for NiTi prealloyed powder, because of the unique modus operandi so as to decrease the cost of shaping, debinding, and sintering process (SDS). The sintering, as a result of the high temperature associated with a long holding time, could transform this indirect process (SDS) into an unsustainable one.

## 3. Results and Discussion

**Figure 4:**
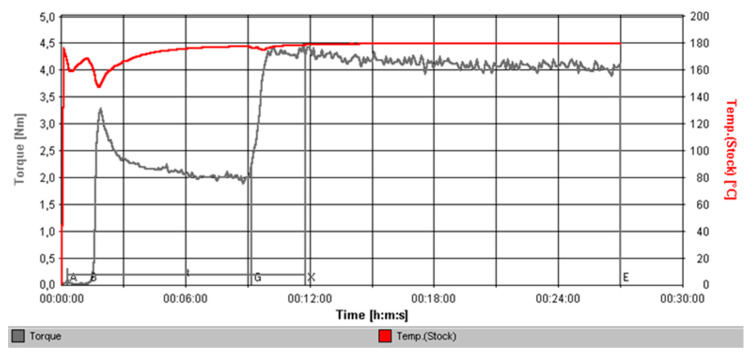
Torque evolution over time during mixing (critical powder volume concentration (CPVC) = 60 vol %).

Filament production was based on a feedstock containing 60 vol % NiTi powder particles and 40 vol % master binder, elastomer (TPE), and plasticizer (P). Figure 4 shows the typical torque evolution over time, and a steady state was attained after 30 min, confirming the homogeneity. A CPVC value of 60 vol % was the best compromise between the NiTi powder particle content and the torque value. The maximum torque value required for the successful extrusion of the examined feedstocks needed to be lower than 5 N·m. For the selected CPVC, the steady-state torque value was 4.2 N·m.

**Figure 5:**
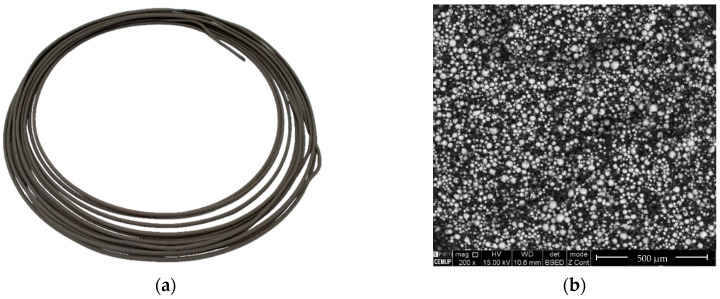
(**a**) Extruded filament of prealloyed NiTi; (**b**) metallic powder particle distribution in the filament.

The NiTi filament was successfully produced with suitable stiffness and flexibility values needed for processing within a 3D printer (Figure 5a). Some predictable geometrical deviations were observed in the filament because of the absence of a calibration system. Nevertheless, the diameter variation did not decrease the printing process quality. In the filament, NiTi powder particles were homogeneously dispersed throughout the binder and additives (Figure 5b).

Tensile and bending tests were performed to confirm the mechanical homogeneity of the filament, and the results were evaluated using the Weibull modulus (*m*). The m value is essential in confirming the reproducibility of the mechanical strength, which is a function of the powder/binder homogeneity of the filament. Values of *m* greater than 10 indicate significant reproducibility, particularly if the test is a flexural test. 

In the tensile test, the ultimate tensile strength was 4.7 MPa and the Weibull modulus (*m*) was 7. However, the bending test revealed that the maximum strength of the filament was 18.5 MPa, with *m* = 23. These results are consistent with the selected tests (tensile and flexural). In powder technologies, the *m* values of the tensile strength are usually lower than those resulting from the flexural strength. In fact, the section subjected to stress in the tensile test is the whole specimen, resulting in values that are significantly lower than those in the bending test, in which the maximum load is applied to only a very thin cross-section of the material. However, despite the lower values of mechanical strength, the analysis of the Weibull modulus revealed a significant level of homogeneity in the filament.

The 0.8 mm nozzle improved the filament flowability, but the dimensional variations and the surface quality of the filament were poor. The use of a nozzle with a diameter of 0.4 mm resulted in some difficulties concerning the filament flowability. The 0.2 mm nozzle produced a material with the best surface quality. However, throughout the process, the nozzle was continuously obstructed. Thus, a nozzle diameter of 0.4 mm was selected. This was the same diameter used in the FFF of another metal study [23]. Parameters related to the nozzle diameter are summarized in Table 2.

For the critical dimensions of the parts processed by SDS, early works on the process of debinding in metallic feedstocks for PEP and PIM have shown that the limit for the third dimension, usually thickness, should be lower than 3 mm. However, because of the development of different binders and environmental atmospheres, this dimension was first increased to 10 mm [26] and then to 15 mm [27]. Nevertheless, this has been studied for PIM, where, during shaping, the green body is subjected to an injection pressure instead of to the atmospheric pressure for a green body shaped by FFF, where the removal of the binder should be easier because of the low compaction of the powder particles. 

**Figure 6:**
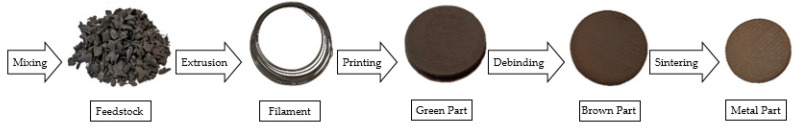
Workflow of fused filament fabrication.

The NiTi green specimen was produced by FFF with Ø20 × 3 (mm) (Figure 6).

**Figure 7:**
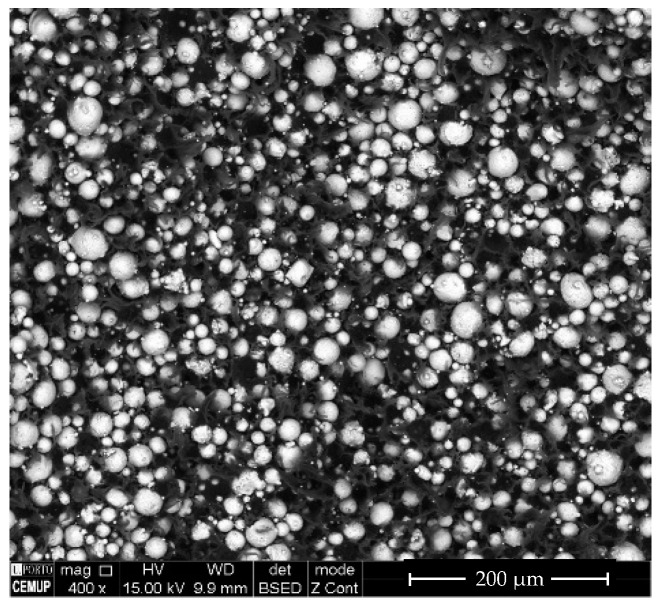
Micrography (SEM) of the green part.

A detailed view of the mixture with binder, additives, and NiTi powder is shown in Figure 7; there is clear homogeneity of the green part.

**Figure 8:**
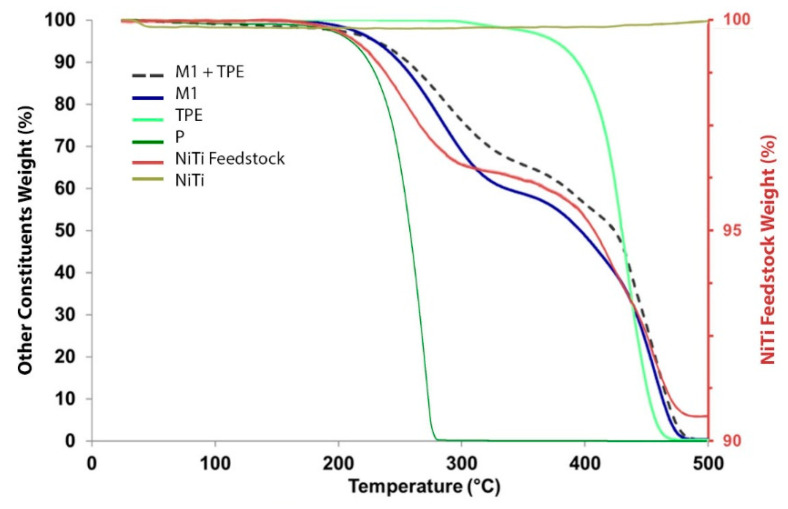
Thermal gravimetric analysis of the binder, additives, and mixed and unmixed NiTi.

The TGA revealed different kinetics for binder (M1) debinding, additives (TPE, P), and the mixture (M1 + TPE / NiTi + M1 + TPE + P) (Figure 8) in a nitrogen atmosphere. Table 3 summarizes the critical temperatures associated with the degradation of different polymer feedstocks.

From the TGA, it is possible to establish the debinding thermal cycle, in which four stages involving temperatures of 100 °C (1 h 30 min), 300 °C (4 h), 500 °C (4 h 30 min), and 600 °C (3 h) must be applied (heating rate = 1 °C min^−1^).

**Figure 9:**
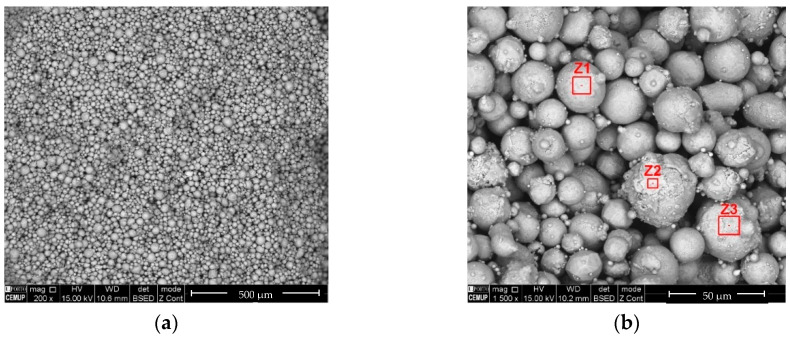
Powder micrographs (**a**) after debinding (SEM); (**b**) selected zones (Z1, Z2, and Z3) for the chemical composition evaluation (EDS).

Figure 9 shows the NiTi powder after debinding. The chemical ratio of Ni:Ti (Table 4) in different powder particles (Z1, Z2, and Z3) of the brown material was nearly constant and was similar to the pristine powder.

The specific surface area of the selected powder was lower than that of the conventionally used PIM powder, which always involves sintering as a primary step in the consolidation process. In FFF technology, the lack of injection pressure must be overcome. Thus, the pressure should be high enough to promote diffusion between powder particles, suitable for increasing sintering kinetics, and to prevent significant agglomeration and chemical surface modifications [28,29].

**Figure 10:**
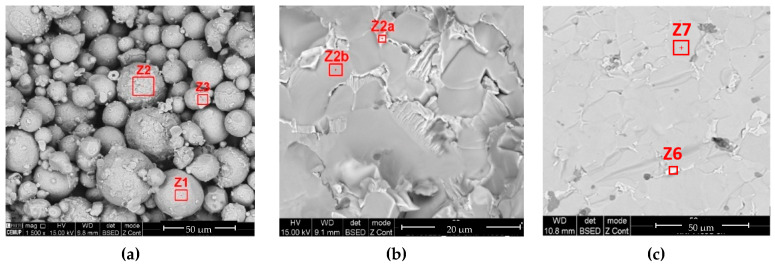
Specimens sintered at (**a**) 1100 °C (1 h), (**b**) 1165 °C (1 h), and (**c**) 1165 °C (5 h).

The sintering process was tested at different temperatures and holding times, and the results revealed that at the lowest temperature (1100 °C) and a holding time of 1 h, sintering was not accomplished. This low-heat thermal cycle produced no sign of pre-sintering (Figure 10a). However, when the maximum temperature was 1165 °C for the same holding time (1 h), sintering was performed efficiently (Figure 10b). Nevertheless, for a holding time of 5 h, the effectiveness of sintering was more evident, and almost full densification was observed (Figure 10c). Therefore, in a H_2_ environmental atmosphere, the best parameter set for the sintering thermal cycle is a maximum temperature of 1165 °C, holding time of 5 h, and heating and cooling rates from 0.1 to 10 K min^−1^. Other sintering studies on NiTi applied high sintering temperatures and holding times from 5 [13,15,28] to 10 h [13,17,30], depending on the size and geometry of the specimens.

**Figure 11:**
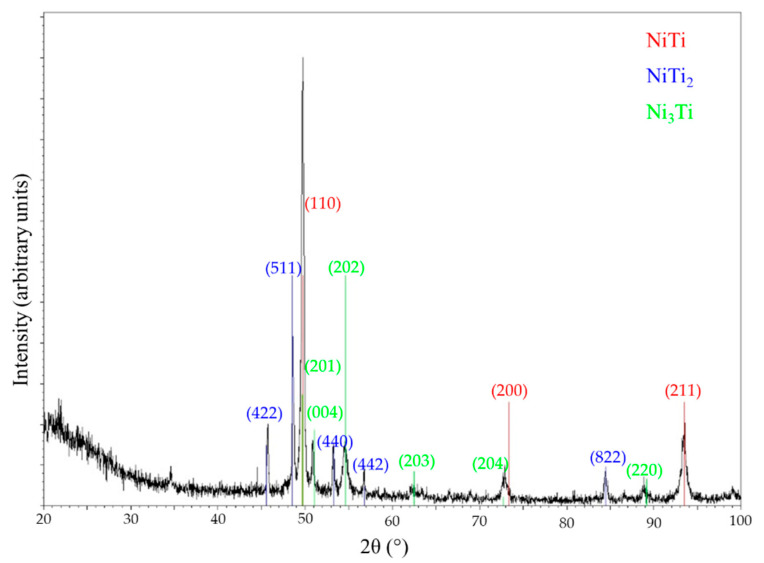
X-ray diffractogram of the NiTi specimens sintered at 1165 °C (5 h).

The Ni:Ti (at %) ratio of the sintered specimens (Table 5) suggests the formation of other phases besides the NiTi phase in the prealloyed powder particles (Table 4). Sintering obliges to high temperatures to promote diffusion. During slow cooling, NiTi_2_ and Ni_3_Ti intermetallic compounds could be formed at the peritectic (984 °C) and eutectic (1116 °C) temperatures [31], respectively. However, the presence of NiTi_2_ as a pristine phase in the prealloyed powder (Figure 1) must be highlighted. In accordance with the results of other authors, as summarized in Table 1, there is no optimal thermal route that is able to produce only a single phase of the SMA NiTi. The specimens sintered at the lowest temperature of 1100 °C (1 h) with a Ni:Ti (at %) ratio of 0.6 suggest the presence of NiTi_2_ as another phase. Specimens sintered at 1165 °C for 1 and 5 h also contain NiTi_2_ (Z2b and Z7) and Ni_3_Ti (Z2a and Z6) in addition to NiTi, according to the intermetallic compounds indexed in the X-ray diffractogram (Figure 11). The phases of NiTi and NiTi_2_ are similar to those obtained by other authors after debinding and sintering the prealloyed powder (Table 1). The presence of Ni_3_Ti was also observed by authors [15], and its presence is due to different atmospheres and/or other heat treatments after sintering.

Table 6 summarizes different hardness values measured in four specimens processed in the same thermal conditions (1165 °C, 5 h) as well as with the mean hardness (MH) and standard deviation (SD). The ultramicrohardness values are higher than the hardness of NiTi (±200 HV) [32,33,34]. This is not attributed to the low load applied during the indentation (40 mN), but to the presence of other intermetallic phases, especially NiTi_2_ and/or Ni_3_Ti, which can potentially increase the hardness values to 700 [35,36] and 742 HV [37], respectively. The elastic moduli and Vickers hardness of the Ni–Ti intermetallic compounds decrease in the following order: Ni_3_Ti > B2_NiTi > B19′_NiTi > NiTi_2_. Ni_3_Ti shows the highest mechanical properties [38]. 

## 4. Conclusions

To address the major production problems for building 3D objects made of NiTi by direct additive manufacturing, particularly those associated with the liquid state, prealloyed NiTi powder particles were processed by an indirect additive manufacturing process. Currently, one of the most promising indirect technologies for additive manufacturing is fused filament fabrication (FFF), which uses maximum processing temperatures that are lower than the melting temperatures of the powder particles. 

According to the available literature, no studies have investigated the application of FFF technology to intermetallic NiTi powder particles. Therefore, it is crucial to have a complete understanding of all five steps involved in the manufacturing process, namely: mixing the constituents of filaments (NiTi powder, binder, and additives), extruding (filament fabrication), printing (shaping the 3D object), debinding (binder removal), and sintering (consolidation of powder particles).

A methodology was developed to evaluate the CPVC when applied to prealloyed NiTi powder filament mixing, and a maximum value of 60 vol % was obtained; this is similar to that of metal injection moulding (MIM) feedstocks, but higher than those of other powder metal filaments. The filament was successfully extruded with a homogeneous distribution of the powder, binder, and additives. The filaments have an excellent level of reproducibility, as evidenced by the high Weibull modulus (*m*) for the filament flexural strength (*m* = 23). It is necessary to account for the absence of pressure when shaping, in contrast to PIM, where the injection pressure can reach higher values.

After shaping different geometries, thermal debinding allows for the total elimination of the binder and additives (T_max_ = 600 °C) in a H_2_ atmosphere. Sintering in a hydrogen atmosphere (T_optimal_ = 1165 °C for 5 h), similar to sintering at higher temperatures, also produces Ni_3_Ti and NiTi_2_ phases. Although secondary phases besides NiTi were formed, FFF appears to be a promising technology for processing NiTi prealloyed powder particles using additive manufacturing.

## Figures and Tables

**Table 1:** Influence of the processing conditions of prealloyed NiTi powders on the final phase composition (shaping = powder injection moulding (PIM)).

Temp (°C)	Holding Time (h)	Atmosphere (Pa)	Post-Processing	Binder/Space Holder (wt %)	CPVC Binder (vol %)	Phases	Ref.
1250	10	Vacuum (1 × 10^−2^)	-	60 Wax 40 polymer	35	NiTi NiTi_2_/Ni_2_Ti_4_O_x_ TiC	[13]
1270	5	Argon Flow	HIP 1050 °C 3 h 195 MPa				
1100	5	Vacuum (-)	Solution treatment 850 °C 1 h Annealing 550 °C 1 h Water quenching	60 Wax 40 polymer	-	NiTi NiTi_2_/Ni_2_Ti_4_O_x_ TiC Ni_3_Ti Ni_4_Ti_3_	[15]
1200							
1270							
1100		Argon Flow (-)					
1200							
1270							
1250	10	Vacuum (-)	-			NiTi NiTi_2_/Ni_2_Ti_4_O_x_ Ni_4_Ti_3_	
1250	10	Vacuum (1 × 10^−3^)	-	PMMA NaCl	20–25	NiTi NiTi_2_/Ni_2_Ti_4_O_x_ TiC	[16]
1250	10	Vacuum (1 × 10^−3^)	-	PE Wax Amide wax NaCl	25–28	NiTi NiTi_2_/Ni_2_Ti_4_O_x_ TiC	[17]
1265							
1260	4	Argon Flow (-)	-	55 Parafin 35 LDPE 10 SA	-	NiTi NiTi_2_/Ni_2_Ti_4_O_x_ TiC Ni_4_Ti_3_	[18]

**Table 2:** Fused filament fabrication (FFF) printer parameters (Ø_nozzle_ = 0.4 mm).

Parameter	Value
Layer height (mm)	0.2
Wall thickness (mm)	0.8
Nozzle temperature (°C)	200
Plate temperature (°C)	50
Print speed (mm s^−1^)	20
Print acceleration (mm s^−2^)	100

**Table 3:** Start and end temperatures of the different debinding steps.

Feedstock Components	1st Stage		2nd Stage	
	Ti (°C)	Tf (°C)	Ti (°C)	Tf (°C)
M1	200	325	360	485
TPE	300	470	-	-
P	175	285	-	-
M1+TPE	200	305	370	490
NiTi	-	-	>300	Light oxidation
NiTi + M1 + TPE + P (feedstock)	150	300	330	490

**Table 4:** Ni:Ti of powder after debinding in the selected zones (Figure 9).

Ni:Ti (at %)		
Z1	Z2	Z3
0.9	1.0	1.0

**Table 5:** Ni:Ti after sintering (Figure 10).

	1100 °C (1 h)			1165 °C (1 h)		1165 °C (5 h)	
	Z1	Z2	Z3	Z2a	Z2b	Z6	Z7
Ni:Ti (at %)	0.6	0.6	0.6	2.6	0.6	2.9	0.6

**Table 6:** Ultramicrohardness of the sintered prealloyed NiTi powder processed by FFF (1165 °C, 5 h).

N° of Specimen	1	2	3	4	MH ± SD
Hardness (HV)	855	939	965	876	887 ± 58
